# Impact of comorbid psychiatric disorders on the outcome of substance abusers: a six year prospective follow-up in two Norwegian counties

**DOI:** 10.1186/1471-244X-6-44

**Published:** 2006-10-20

**Authors:** Anne Signe Landheim, Kjell Bakken, Per Vaglum

**Affiliations:** 1 Centre for Addiction Issues, Department for Substance Abuse, Innlandet Hospital Trust, Norway; 2 Department of Behavioural Sciences in Medicine, Faculty of Medicine, University of Oslo, Norway

## Abstract

**Background:**

Most help-seeking substance abusers have comorbid psychiatric disorders. The importance of such disorders for the long-term course of substance abuse is, however, still unclear. The aim of this paper is to describe six-year outcomes regarding death and relapse among alcoholics and poly-substance abusers and to analyse the predictive value of lifetime psychiatric disorders on relapse.

**Methods:**

A consecutive sample of substance-dependent patients who received treatment in two counties in Norway (n = 287) was followed up after approximately six years. Information on socio-demographics, Axis I (CIDI) and II disorders (MCMI-II) and mental distress (HSCL-25) was gathered at baseline. At follow-up, detailed information regarding socio-demographics, use of substances (AUDIT and DUDIT) and mental distress (HSCL-25) was recorded (response rate: 63%).

**Results:**

At six-year follow-up, 11% had died, most often male alcoholics (18%). Among the surviving patients, 70% had drug or alcohol related problems the year prior to follow-up. These patients were, classified as "relapsers". There were no significant differences in the relapse rate between women and men and among poly-substance abusers and alcoholics. The relapsers had an earlier onset of a substance use disorder, and more frequently major depression and agoraphobia. Multivariate analysis indicated that both psychiatric disorders (major depression) and substance use factors (early onset of a substance use disorder) were independent predictors of relapse.

**Conclusion:**

For reducing the risk of long-term relapse, assessment and treatment of major depression (and agoraphobia) are important. In addition, we are in need of a comprehensive treatment and rehabilitation program that also focuses on the addictive behaviour.

## Background

Most help-seeking substance abusers have comorbid symptom disorders and/or personality disorders [1-3]. The importance of such disorders for the long-term course of substance abuse is, however, still unclear. Most studies to date have examined the relationship between psychiatric disorders and substance use outcomes only in short-term follow-up studies [4,5]. The few long-term studies (> 5 years) of comorbid disorders among cocaine abusers [6], opiate abusers [7-11] and alcoholics [12-15] are mainly from single treatment institutions and examine samples with alcoholics or drug addicts only, or males and females separately [4]. Consequently, there is a need for outcome studies in clinically representative samples that differentiate between genders as well as between alcoholics and poly-substance abusers, and that include both measures of substance abuse and Axis I (symptom disorders) and Axis II (personality disorders) disorders. By controlling for socio-demographic and substance use factors, such studies may clarify whether lifetime Axis I and II disorders have a unique and independent impact on the long-term course of substance abuse. If they have such an impact, it may have important implications for treatment planning [16].

We have conducted a six-year prospective study of a consecutive sample of patients who received treatment for substance abuse in two counties in Norway between 1997 and 1998 (n = 287). At baseline, extensive information about socio-demographics, substance use factors, psychiatric disorders (Axis I and II disorders) and mental distress were gathered through personal interviews and self-reports. At follow-up, detailed information about socio-demographics, substance use and mental distress was recorded through a postal questionnaire. "Relapsers" were defined as persons with drug- or alcohol-related problems at both follow-up and over the preceding year. Baseline variables were analysed as predictors of "relapse".

This paper explores the following research questions:

1. What is the frequency of death and relapse at the six-year follow-up in the total sample, as well as among the sub-groups of poly-substance abusers and alcoholics? Do possible gender differences exist in and between the two sub-groups?

2. Is there any difference between abstainers and relapsers with regard to socio-demographic variables, substance use variables and Axis I and II disorders? Are there differences within poly-substance abusers and alcoholics?

3. Are lifetime Axis I and II disorders assessed at baseline independent predictors of relapse at follow-up, when controlling for substance use variables and socio-demographic variables?

## Methods

### Sampling

A consecutive sample (n = 287, 73% male, mean age 38.6 ± 11.3 years) of DSM-IV substance-dependent patients (156 alcoholics and 131 poly-substance-dependent) from three outpatient (n = 157) and six inpatient (n = 130) public substance abuse facilities in two Norwegian counties were recruited from September 1997 to November 1998. These facilities were owned or financed by the two counties. In the six-inpatient units, there were two therapeutic communities, one shelter, one short-term unit (6 weeks) and two long-term units (between 3 months and 11/2 year). Two of the inpatient units were for males, and one of the units was for females. The therapeutic communities offered treatment to young drug addicts. The programs had abstinence and rehabilitation as their primary goal and they offered both individual and group therapy. Few of the programs assessed and specifically treated diagnosed psychiatric disorders. Treatment in most of the programmes was not based on a specific ideology or philosophy. The three outpatient units offered primarily individual therapy and the main goal for treatment was abstinence and rehabilitation.

The participation rate was 42% (287/690). Those who were recruited and those who did not participate did not differ significantly with regard to socio-demographic variables and substance use variables except for age. Our sample was somewhat older (38.6 vs. 35.6; p < 0.001). To evaluate further whether our sample was representative, we compared our sample with a national sample (n = 5,000), drawn from the nationwide treatment system in Norway (17). Our sample was older (23% vs. 36% were younger than 30 years, p < 0.001), more often married/cohabiting (36% vs. 27%, p = 0.02), and more frequently using alcohol (63% vs. 50%, p < 0.001), and less frequently using heroin (16 vs. 21%, p < 0.05). In general, the sample appeared somewhat skewed towards having fewer young drug addicts compared with the national sample. Further information about sampling, subjects, and methods has been described elsewhere (3). All patients gave written informed consent to assessment at admission and to be contacted at a six-year follow-up.

A follow-up study of the same sample (n = 287) was conducted on average 75 months (SD = 5.0) later (July to November 2004). At this time, 33 patients (11%) had died. Attempts were made to contact the remaining patients (n = 254) by telephone to inquire if they wanted to participate in the follow-up study. A questionnaire was then mailed to them. They received 300 Norwegian kroner for completing the form (approximately 38 EURO). In all, 160 patients (63% of the surviving participants) answered the questionnaire. Of the group who did not respond (n = 94), 14 refused participation, 23 were not located, and 57 received the assessment form twice (according to instructions from the Norwegian Data Inspectorate) but did not answer it. When including the deceased patients, the participation rate was 67% (193/287). In the analyses, living and deceased patients were analysed separately.

When comparing the participants (n = 160) with the non-participants (n = 94), there were no significant differences with regard to socio-demographics, substance use variables, Axis I and II disorders, mental distress (HSCL-25), inpatient or outpatient status, and treatment history at baseline.

### Subjects

At baseline, all subjects met DSM-IV criteria for current substance dependence or abuse. Fifty-four percent (n = 156) of the subjects were classified as pure alcoholics, i.e. a diagnosis of alcohol dependence with no other drug- dependence or abuse. Forty-six percent (n = 131) were classified as poly-substance abusers, all being dependent on illicit drugs. Of these, 85% used heroin and/or amphetamine as the primary substance of abuse. In addition, 35% of the poly-substance abusers fulfilled the criteria for current alcohol dependence or abuse. The mean number of illicit drugs on which poly-substance abusers were currently dependent was 3.9 (SD = 2.1).

### Measures at baseline

The patients were assessed using the Norwegian National Client Assessment form [17] (socio-demographic, treatment history), the Composite International Diagnostic Interview (CIDI) [18] (lifetime and current Axis I disorders, including both psychiatric disorders and substance use disorders), and two self-report questionnaires: the Millon Clinical Multiaxial Inventory (MCMI-II) [19] (Axis II disorders) and the Hopkins Symptom Checklist 25 (HSCL-25) (mental distress) [20].

A diagnosis for substance abuse, harmful use or dependence was assessed using the CIDI (based on DSM-IV and ICD-10 criteria). The CIDI was also used to classify the patient's main substance of abuse as either alcohol dependence with no other drug dependence or abuse, or as poly-substance dependence. In addition, the patients reported the number of alcohol units used on a typical drinking day during the last 12 months.

The CIDI interviews were primarily conducted by the two researchers (K.B. and A.L.) who took part in the training workshops offered by The National WHO Training Centre in Munich. One third of the interviews for this study were conducted by trained clinicians (psychiatrists, social workers, psychiatric nurses), who had been trained by K.B. and A.L. in a standardised 4-day training program. The CIDI has shown good feasibility in general populations, high inter-rater reliability and has been subjected to tests of reliability and validity with satisfactory results [21].

Findings from several studies of the psychometric properties of the MCMI-II report acceptable test-retest reliability and generally acceptable levels of convergent and discriminant validity of the scales [22]. In the baseline study, the HSCL-25 showed excellent reliability with a Cronbach's alpha of 0.94.

### Measures at follow-up (postal questionnaire)

The follow-up questionnaire covered socio-demographics and treatment history using the same questions that were used at baseline (The Norwegian National Client Assessment form).

Due to a lack of resources, we did not have the opportunity to conduct personal interviews (i.e.- the CIDI) to make psychiatric diagnoses and evaluate substance use disorders at follow-up. In stead, we used well-established self-report instruments (AUDIT, DUDIT and HSCL-25).

Mental distress during the week immediately prior to assessment was measured using the HSCL-25 (as at baseline). Reliability according to Cronbach's alpha for HSCL-25 at follow-up was 0.96.

To measures substance use we used the Alcohol Use Disorders Identification Test (AUDIT) [23] and the Drug Use Disorders Identification Test (DUDIT) [24]. Both are screening instruments for identifying persons with a problematic use of substances during the previous 12 months. Both instruments are standardized and based on selected criteria for substance abuse, harmful use and dependence according to the ICD-10 and DSM-IV diagnostic systems.

The AUDIT core questionnaire consists of 10 items: three questions on quantity and frequency of drinking, three items on alcohol dependence and four questions on problems caused by drinking. Weighted scoring with respect to the frequency or the time of occurrence comprises a total score that ranges from 0 to 40. Standard instructions state that a total score of eight or more for men and six or more for women indicates a strong likelihood of hazardous alcohol consumption. In a review of the AUDIT by Reinert and Allen (2002) [25], the median sensitivity for identifying individuals with alcohol dependence or misuse was 0.86 and the median specificity was 0.89. In our study, reliability according to Cronbach's alpha was 0.93.

DUDIT is an 11-item self-report instrument intended for use together with the AUDIT. The DUDIT's cut-off points for drug-related problems are scores of six or more for men and two or more for women out of a maximum of 44 points. The psychometric properties of the DUDIT were evaluated in a sample of heavy drug users from prison, probation, and in a general Swedish population sample. In the drug users sample, the DUDIT predicted drug dependence with a sensitivity of 90% for both DSM-IV and ICD-10 criteria and a respective specificity of 78% and 88% [24]. In our study, reliability according to Cronbach's alpha was 0.95.

In addition to the AUDIT and DUDIT, the questionnaire also asked: 1) How many months have you been totally abstinent from any substances from baseline to follow-up? And 2) How many alcohol units did you use on a typical drinking day in the last 12 months before assessment?

Data of deceased patients were provided by the National Register of Demographical Data. Death certificates (causes of death) were obtained from the Cause of Death Registry (Division for Health Statistics in Statistics Norway).

The study protocol at both baseline and follow-up was reviewed and approved by the Regional Committee for Medical Research Ethics and by the Norwegian Data Inspectorate.

### Dependent variable: abstainer or relapser

Subjects were classified into one of two groups, abstainers or relapsers, based on AUDIT and DUDIT scores. The "abstainers" (n = 48) consisted of persons without any drug- or alcohol-related problems at follow-up and during the 12 months prior to follow-up (based on an AUDIT score below eight points for men and six points for women and a DUDIT score below six for men and two for women). The "relapsers" (n = 112) consisted of persons with drug- or alcohol-related problems during the year prior to and at follow-up (based on an AUDIT score ≥ 8 for men and ≥ 6 for women, or a DUDIT score ≥ 6 for men and ≥ 2 for women).

### Independent variables

Potential risk factors assessed at baseline were selected according to the literature [5]. They consisted of: socio-demographics (age, sex, marital status and employment status), substance use variables (main substance of abuse, age at onset of a substance use disorder, duration of a substance use disorder and mean number of lifetime substance use disorder diagnoses) and psychiatric disorders (any lifetime Axis I disorders, number of lifetime Axis I disorders, single lifetime Axis I disorders, any personality disorders, number and type of personality disorders).

Lifetime instead of current Axis I disorders were selected as a possible predictors of relapse. The reason for this choice was that lifetime Axis I disorders and current Axis I disorders were highly correlated. In addition, current Axis I disorders seem to be more related to the situation at assessment and we assume that lifetime disorders are more valid with regard to the disposition for Axis I disorders. Results by current Axis I disorders are presented in the results section but were not examined as potential predictors of relapse.

### Statistical analysis

The data were analysed in two steps. First, univariate comparisons were made between groups using chi-square tests for categorical data, independent t-tests for continuous variables and paired t-tests for continuous variables at baseline vs. follow-up. Second, a prognostic strategy was used to analyse the predictive value of lifetime psychiatric disorders on relapse at follow-up. A logistic regression model was used to identify the odds ratio of all explanatory variables (covariates) that were significant in the univariate analyses. The dependent variable in the regression model was the substance use outcome at follow-up (relapser = 1, abstainer = 0). A correlation analysis was done between the major covariates used in the model. If covariates were highly correlated (r > 0.40), we selected one of them for the model. We used a backward elimination procedure in the logistic regression model and checked the goodness of fit via chi-square using the Hosmer and Lemeshow goodness-of-fit test [26]. All analyses were conducted using SPSS for Windows, version 11.0 (SPSS 2005).

## Results

### Patients who died

At the six-year follow-up, 11% of the original sample had died (33/287); information regarding causes of death was obtained for 26 patients. In 21 of the patients, death was substance-related (overdose, poisoning, alcoholic cirrhosis of the liver, toxic effect of alcohol). Among the remaining five patients, two died from cancer, one from suicide and for two patients the cause of death was unknown.

Selected baseline characteristic measures were used to compare the 160 surviving persons and the 33 deceased persons (Table 1). At baseline, the since-deceased patients were older (44.2 vs. 38.7 years, p = 0.01), more frequently alcoholics (73% vs. 54%, p = 0.04) and more likely to have a later onset of a substance use disorder (mean age of onset, SUD: 26.4 vs. 22.3 years, p = 0.03).

**Table 1:** Sample characteristics at baseline in surviving and deceased patients (N = 193).

	**Surviving patients (N = 160)**	**Deceased patients (N = 33)**	**p-value**
Female, %	29	18	0.19
Age, mean years (SD)	38.7 (11.0)	44.2 (10.1)	0.01
Employment, %	34	29	0.60
Marital status, %	39	30	0.37
Alcoholics, %	54	73	0.04
Onset of substance use disorder, mean years (SD)	22.3 (9.9)	26.4 (12.5)	0.03
Duration of substance use disorder, mean years (SD)	16.4 (9.1)	17.7 (9.1)	0.45
Lifetime Axis 1 disorder, %	91	82	0.13
Lifetime anxiety disorders, %	81	81	0.95
Lifetime affective disorders, %	64	52	0.19
Any personality disorder, %	72	73	0.90

Male alcoholics had the highest percentage of deaths (n = 22/121, 18%) compared to female alcoholics (n = 2/35, 6%) and female (n = 4/44, 9%) and male (n = 5/87, 6%) poly-substance abusers (p = 0.05). The mean age of male alcoholics when they died was 51.5 years old (SD = 10.6), while it was 49.5 years (SD = 9.1) in female alcoholics. In deceased male poly-substance abusers, the mean age was 39.5 years (SD = 3.5) while deceased female poly-substance abusers had a mean age of 38 years (SD = 9.2) at the time of death. The age difference at death between alcoholics and poly-substance abusers was significant (38 vs. 51, p = 0.01).

The observed death rate each year for the total sample is 1.9%, 3% among male alcoholics, 1% in female alcoholics, 1% in male poly-substance abusers and 1.5% among female poly-substance abusers.

### Substance use outcome among the surviving patients

Of the surviving patients who participated at follow-up, 30% (48/160) were abstainers and 70% (112/160) had relapsed according to their AUDIT and DUDIT scores. The mean score on the AUDIT for abstainers was 2.2 (SD = 2.5) and the mean score for the DUDIT was 0.33 (SD = 0.9). The mean number of months of total abstinence from any substances between baseline and follow-up was 53.2 months (SD = 27.5), with a median of 57 months. Fifty-two per cent (n = 25) of the abstainers had been totally abstinent from any substances for five years or more.

Seventy per cent were relapsers (n = 112) at follow-up as well as over the previous year. The mean AUDIT score for relapsers was 18.2 (SD = 8.6) and the mean DUDIT score was 16.9 (SD = 9.8). The mean number of months of total abstinence from any substances between baseline and follow-up was 20.9 months (SD = 20.8), with a median of 14 months. From baseline to follow-up 43% (n = 48) of the relapsers had been totally abstinent for 11 months or less.

At both baseline and follow-up, the patients reported the number of alcohol units used on a typical drinking day in the last 12 months. When comparing the mean units of alcohol used at baseline and follow-up in abstainers (paired sample t-test), we found a significant decrease from 16.6 (± 8.5 (SD) alcohol units to 7.0 (± 4.4) alcohol units (p = 0.001). In relapsers, we found no significant decrease in the use of alcohol from baseline to follow-up (mean number of alcohol units used on a typical drinking day = 18.3 units ± 11.8 vs. 16.3 units ± 13.0, p = 0.28).

There were no significant gender differences in and between poly-substance abusers and alcoholics with regard to relapse rate at follow-up (female alcoholics, 24%; male alcoholics, 32%; female poly-substance abusers, 35%; male poly-substance abusers, 27%, p = 0.80).

Among the poly-substance abusers, 28 received methadone treatment during the follow-up period and at follow-up. There was no significant difference in relapse rate between poly-substance abusers with or without methadone treatment (79% vs. 65%, p = 0.22). Relapsers on methadone treatment were using mainly illicit drugs rather than alcohol.

### Comparisons of abstainers and relapsers

Table 2 shows that the relapsers were younger (37.7 years vs. 41.2 years, p = 0.06) and less often married or cohabiting at both baseline (33% vs. 51%, p = 0.04) and follow-up (31% vs. 54%, p = 0.01) as, compared to abstainers. Additionally, relapsers differed significantly from abstainers by having an earlier onset of a substance use disorder (52% vs. 31%, onset SUD before the age of 18, p = 0.01).

**Table 2:** Socio-demographic variables (A), substance abuse variables (B) treatment variables (C) at baseline (T1) and follow-up (T2) between abstainers and relapsers.

**A. Socio-demographic variables**	**Abstainers N = 48**	**Relapsers N = 112**	**p-value**
Sex, female, T1, %	29	30	0.97
Age, mean years (SD), T1	41.2 (11.8)	37.7 (10.6)	0.06
Employment, %			
T1	38	32	0.48
T2	35	26	0.22
Marital status, married or cohabiting, %			
T1	51	33	0.04
T2	54	31	0.01

**B. Substance abuse variables**	**Abstainers N = 48**	**Relapsers N = 112**	**p-value**

Poly-substance abusers, T1, %	46	46	0.94
Onset of SUD, < 18 years, T1 %	31	52	0.01
Duration of SUD, ≥ 15 years, T1 %	50	59	0.29
Mean number of lifetime SUD diagnoses (abuse and dependence), T1	2.2 (1.6)	2.6 (2.1)	0.25

**C. Treatment variables**	**Abstainers N = 48**	**Relapsers N = 112**	**p-value**

In-patients facilities, T1, %	52	56	0.63
Out-patient facilities, T1,%	48	44	
Treatment in the substance abuse field, prior to T1, %	60	72	0.14
Treatment in the mental health care system, prior to T1, %	36	39	0.75
Treatment in the substance abuse field, between T1 and T2, %	54	75	0.01
Treatment in the mental health care system, between T1 and T2, %	40	55	0.06

There were no significant differences between abstainers and relapsers on whether they received treatment in outpatient or inpatient facilities at baseline (52% vs. 56%, p = 0.63). The relapsers had more often received treatment in the substance abuse field (75% vs. 54%, p = 0.01) and in the mental health care system (55% vs. 40%, p = 0.06) between baseline and follow-up, compared to abstainers.

With regard to comorbid psychiatric disorders, Table 3 shows that lifetime Axis I disorders were more common among relapsers as compared to abstainers (94% vs. 83%, p = 0.03). In particular, relapsers had higher rates of major depression (51% vs. 34%, p = 0.05), agoraphobia (50% vs. 33%, p = 0.05) and a higher number of Axis I disorders (mean number: 3.9 vs. 2.8, p = 0.01). Any current Axis I disorders were significantly more frequent among relapsers than abstainers (88% vs. 75%, p = 0.04). Furthermore, current major depression tended to be more frequent among relapsers than abstainers (40% vs. 29%, p = 0.18). Results regarding current Axis I disorders had the same direction as lifetime disorders but given the smaller number of patients with a current disorder the results did not reach significance.

**Table 3:** Lifetime Axis I disorders and mental distress at follow-up between abstainers and relapsers.

**Axis I disorders**	**Abstainers N = 48**	**Relapsers N = 112**	**p-value**
Any Axis I disorder, %	83	94	0.03
Affective disorders, %	55	67	0.15
Major depression, %	34	51	0.05
Dysthymia, %	30	39	0.28
All anxiety disorders, %	75	84	0.19
*Agoraphobia, %	33	50	0.05
Social phobia, %	40	51	0.20
Simple phobia, %	40	48	0.39
Post traumatic stress disorder, %	21	22	0.89
Somatization disorder, %	28	28	0.99
Number of Axis I disorders, mean (SD)	2.8 (2.0)	3.9 (2.7)	0.01
Mental distress, HSCL-25, mean (SD) T2	1.52 (0.5)	2.17 (0.5)	0.001

*Agoraphobia with and without panic attack

Relapsers also had a significantly higher mental distress score at follow-up compared with abstainers (mean score on HSCL-25: 2.17 vs. 1.52, p = 0.001). There were no significant differences between relapsers and abstainers with regard to frequency and type of personality disorders (Table 4).

**Table 4:** Lifetime Axis II disorders between abstainers and relapsers. MCMI-II, Base rate score ≥ 85.

**Axis II disorders**	**Abstainers N = 48**	**Relapsers N = 112**	**p-value**
Any personality disorder, %	71	73	0.84
Paranoid, %	2	5	0.48
Schizoid, %	11	19	0.25
Schizotyp, %	7	16	0.12
Antisocial, %	30	32	0.73
Borderline, %	25	34	0.26
Histrionic, %	11	11	0.99
Narcissistic, %	11	13	0.74
Avoidant, %	30	42	0.16
Dependent, %	21	17	0.63
Compulsive, %	9	4	0.19
Passive-aggressive, %	39	40	0.88
Self-defeating, %	18	30	0.15
Aggressive- Sadistic, %	18	25	0.38
Number of Axis II disorders, mean (SD)	2.3 (2.0)	2.8 (2.7)	0.25

When doing the analyses separately among alcoholics and poly-substance abusers, the only difference between the two subgroups was that relapsers had an earlier onset of a substance use disorder compared to abstainers in poly-substance abusers (81% vs. 50%, p = 0.01, onset before 18 years old) but not in alcoholics (27% vs. 15%, p = 0.25).

### Independent predictors of relapse at follow-up

To identify whether lifetime psychiatric disorders had an independent impact on being a relapser at follow-up, a logistic regression analysis was conducted. The dependent variable was relapser = 1, abstainer = 0. As independent variables, we included those baseline variables that were significant or nearly significant in univariate analyses in Tables 2, 3 and 4 (age, marital status, onset of a substance use disorder, lifetime major depression and agoraphobia). Age was excluded from further analyses because it was highly correlated with onset of a substance use disorder (r = 0.61). The following covariates were included in the model: marital status (1 = married/cohabited and 0 = not married/cohabited), age at onset of a substance use disorder (1 = below 18 years and 0 = 18 years or older), major depression (1 = yes and 0 = no) and agoraphobia (1 = yes and 0 = no). Next, we used a backward elimination procedure in the logistic model and checked the goodness of fit using chi-square via the Hosmer-Lemeshow test. We excluded agoraphobia and marital status from the model because the goodness-of-fit test was higher by including only major depression and onset of a substance use disorder (Hosmer-Lemeshow: chi-square = 3.52, df = 8, p = 0.89 vs. chi-square = 0.02, df = 2, p = 0.95).

In Table 5, the final model shows that there are two significant independent predictors of being a relapser at follow-up: an early onset of a substance use disorder (OR = 2.3, p = 0.02) and the occurrence of a lifetime major depression (OR = 2.1, p = 0.05). The two predictors remained significant after controlling for gender and main substance of abuse (alcoholics vs. poly-substance abusers).

**Table 5:** Predictors of relapse at six-year follow-up. Odds ratio (OR) and 95% confidence intervals (CI) (n = 160).

**Predictors**	**OR**	**95% CI**	**p-value**
Onset of SUD, before 18 years (0 = no, 1 = yes)	2.3	1.14–4.96	0.02
Major depression (0 = no, 1 = yes)	2.1	1.10–4.51	0.05

Hosmer-Lemeshow goodness-of-fit test; chi-square = 0.02, df = 2, p = 0.95

## Discussion

### Outcome at six-year follow-up

By the six-year follow-up, 11% had died, most of whom were male alcoholics. In two-thirds of these deaths, the cause of death was substance-related. The yearly death rate of approximately 2% is quite similar to other studies of substance-dependent persons [27-30], and higher than in the general population in Norway in 2004 (0.9%). The yearly death rate in the sample is higher both for women (1.3%, mean age at T1: 36 years) and men (2.1%, mean age at T1: 39) compared with yearly death rates among women (0.06%) and men (0.1%) in the general population between 35–39 years [31]. The observed death rate each year for male alcoholics was approximately 3% compared to 0.4% among men in the same age group (50–54 years) in the general population in 2004. Our findings indicate that chronic alcohol dependence in men may lead to premature death, and the importance of this may be overlooked in short-term studies.

Among surviving patients, 70% (n = 112/160) still had drug- or alcohol-related problems at follow-up and over the year immediately prior to follow-up (relapsers). In relapsers, there was no significant decrease in use of alcohol between baseline and follow-up.

Studies with comparable follow-up intervals (5–8 years) have reported relapse rates in alcoholics that lie between 45% and 75% [11,12,29,32-37] and in illicit drug abusers (mainly opiates) between 39% and 84% [6,8,30,38-44]. In many studies, including ours, it is between 60% and 70%.

There were no significant differences between poly-substance abusers and alcoholics with regard to relapse rate. In different studies the relapse rate usually are higher among poly-substance abusers than among alcoholics [4]. However, more alcoholics died in the current study.

Methadone programs started after 1998 in Norway. The main goal of methadone treatment was to eradicate the use of heroin and other illicit drugs. One would expect to find that patients on methadone treatment (n = 28) were more abstinent at the six-year follow-up than poly-substance abusers without such treatment. The results are not in the expected direction (79% vs. 65%, p = 0.22).

There are few studies comparing relapse rate between female and male substance abusers within the same sample. A recent review concluded that women had treatment outcomes that were as good as or better than those of men [45]. In the present study, there was no significant difference in relapse rate between women and men.

### Predictors of relapse: socio-demographic and substance abuse variables

The relapsers were younger, more seldom married or cohabiting and had an earlier onset of a substance use disorder. These findings concur with the literature [5,6,15,30,34].

An early onset of a substance use disorder had an independent impact on being a relapser at follow-up, after controlling for main substance of abuse (alcoholics and poly-substance abusers) and Axis I disorders. This is in accordance with other studies showing that an early onset of a substance use disorder often leads to a poor substance use outcome and is a valid measure of severity of substance abuse [5].

The early onset of a substance use disorder may lead to the rapid progression of abuse and dependence and social disintegration, and the way back to social integration and sobriety may be more difficult. To become more socially integrated and reduce the risk of relapse, such persons are in great need of psychosocial treatment and the availability of good rehabilitation programs.

### Predictors of relapse: comorbid psychiatric disorders

Concerning Axis I disorders, relapsers had more lifetime Axis I disorders and especially more major depression and agoraphobia (assessed at baseline). Subjects with a lifetime major depression were significantly more likely to be relapsers at six-year follow-up, even after controlling for socio-demographic factors, agoraphobia and substance use variables. These results were significant both in alcoholics and in poly-substance abusers.

The predictive value of major depression on relapse in long-term studies has not been consistent in either studies of alcoholics or illicit drug abusers. Different characteristics of the samples, different measures and additional variables such as further treatment or social network and support may influence the relationship between major depression and relapse.

One long-term follow-up study of alcoholics [30] found that depression predicted heavier alcohol consumption at follow-up but only among women. Another study [46] found that depression and drinking outcomes were significantly related at the three-year follow-up. Furthermore, Kranzler et al. (1995) [47] found that major depression was associated with lower intensity of drinking at three-year follow-up. Thus, our data regarding alcoholics are supported by other studies.

In long-term studies of illicit drug abusers, Verthein et al. (2005), [9] showed in their four-year follow-up study of opiate-dependent patients that lifetime diagnosis of mental disorders had no prognostic relevance for the long-term course of drug dependency. In another five-year follow-up study of opiate users, depression predicted less drug use in the year preceding follow-up, whereas greater hostility predicted increased drug use [7]. Ravndal and Vaglum (1998) [11] found in their five-year follow-up study of drug abusers that personality disorders and psychopathology did not directly predict substance use outcome. Except for our study and the study by Verthein, these studies are from single treatment institutions and not from a heterogenic sample derived from a catchment area. Our findings concerning illicit drug abusers, therefore, need replication in further studies of clinically representative samples.

Major depression is a frequent disorder among treatment-seeking substance abusers. In our sample, 46% fulfilled the criteria for such a disorder and there was no significant difference between rates among poly-substance abusers and alcoholics. In 68% of those with a lifetime major depression, the disorder occurred for the first time at least one year *after *the debut of a substance use disorder (secondary depressive disorder). There was, however, no significant difference in relapse rate between subjects developing major depression before or after the onset of a substance use disorder. Consequently, both primary and secondary major depressions should be addressed in treatment.

Relapsers with a lifetime major depressive disorder assessed at baseline had a higher mental distress score at follow-up compared to relapsers without such a disorder (mean score on HSCL-25 = 2.28 ± 0.57 (SD) vs. 2.07 ± 0.55, p = 0.06). Also, 63% of relapsers with a lifetime major depression had received treatment in the mental health care system between baseline and follow-up, compared to 50% in relapsers without a lifetime major depression (p = 0.18). These findings support the notion that the lifetime major depression identified at baseline indicates a chronic condition that continues to influence the patient into the future. Consequently, it is important to address simultaneously both depression (and agoraphobia) and the addictive behaviour in the same programme [48]. Both pharmacological and behavioural interventions have been shown to increase abstinence rates in patients with combined depression and substance use disorders [49]. Unfortunately, patients with both psychiatric disorders and a substance use disorder often receive treatment from two parallel treatment systems [48].

A recent review of personality disorders and substance abuse indicated that the course and outcome of substance abuse was very often directly and negatively related to the presence of one or more personality disorders [50]. Our results do not confirm such a relationship, neither in univariate nor in multivariate analysis. In the cited review [48], however, nearly all of the studies were short-term (6 months-2 years). The question of the long-term impact of personality disorders on substance abuse needs further research.

### Strength and limitations

The strength of the current study is the consecutive sample based on a treatment population, the heterogeneity of treatment institutions included and the heterogeneity of the sample by including both poly-substance abusers and alcoholics. The use of structured interviews and standardized rating scales and self-report instruments with good and well established reliabilities are also strengths.

The study has some limitations. As in all clinical studies, it is unclear how representative the sample is and how context-dependent the results are. The study population is treatment seeking substance abusers receiving treatment in the substance abuse field in two counties in Norway. Our response rate of 63% is acceptable for a long-term follow-up study. There were no significant differences with regard to socio-demographics, substance use variables, Axis I and II disorders, mental distress, in-patient or outpatient status, and treatment history assessed at baseline between the participants in the follow-up study (n = 160) and the refusers at follow-up (n = 94). It is hard to say whether those who did not participate in the follow-up study have relapsed or remained unimproved. We know that 24% of the dropouts were not located which means that they had no stable residence. This may indicate that they are unimproved and that the relapse rate is underestimated.

Although the response rate at 6-year follow-up is acceptable, the 42% response rate at intake was lower than desired and the sample appeared somewhat skewed towards having fewer young poly-substance abusers compared with a national sample [17]. Further, we found in the follow-up study that relapsers were younger than abstainers. A higher amount of young drug addicts in both the index-sample and the follow-up sample may have increased the relapse rate.

Other factors besides personal characteristics at baseline may also influence relapse, such as important life events, treatment received and social stability factors. Also, we were not able to examine stability or temporal changes in use of substances. Unfortunately, we did not have any information about the nature of treatment or the patients' compliance with treatment. Because of the long-term follow-up period, it is difficult to analyse the effect of treatment received at index-admission on substance use outcome. Many of the patients had received treatment in the substance abuse field and the mental health care system both before and after baseline.

The validity of recall and self-report remains a major problem in this type of research, especially in long-term studies. Different studies have showed that self-reports in substance abusers may constitute a valid instrument for assessing substance abuse outcome [51]. In the present study, we have used standardized instruments for assessing substance use outcome. At baseline, we assessed diagnoses for abuse and dependence by using a structured interview (CIDI). At follow-up, we used screening tools for assessing dependence and abuse (AUDIT and DUDIT). These screening tools have shown a high sensitivity and specificity for identifying abuse and dependence. In addition, the patients' answers about use of substances will not have any influence on their possibility of receiving treatment or other social welfare goods.

## Conclusion

In conclusion, at six-year follow-up 11% of the participants had died, with male alcoholics having the highest percentage of deaths (18%, n = 22). Among surviving patients, 70% (n = 112) had relapsed. There were no significant differences in the relapse rate between women and men or between poly-substance abusers and alcoholics. Relapsers had significantly more lifetime Axis I disorders, especially major depression and agoraphobia. Multivariate analysis indicated that lifetime major depression and an early onset of a substance use disorder were independent predictors for relapse, both among alcoholics and among poly substance abusers. For reducing the long-term risk of relapse, the assessment and treatment of major depression (and agoraphobia) in substance abusers is important. Furthermore, we are in need of a comprehensive treatment and rehabilitation program that also focuses on the addictive behaviour.

## Competing interests

The author(s) declare that they have no competing interests.

## Authors' contributions

AL have carried out conception and design, data collection, data analysis, interpretation of data and drafted the manuscript. KB has carried out conception and design, data collection, data analysis, interpretation of data and has been involved in revising the manuscript and given final approval of the version to be published. PV has contributed to conception and design and has contributed in the drafting, as well as having been involved in revising the manuscript and given final approval of the version to be published. All authors read and approved the final manuscript.

## Pre-publication history

The pre-publication history for this paper can be accessed here:


